# Chemical Systems with Limit Cycles

**DOI:** 10.1007/s11538-023-01170-3

**Published:** 2023-07-04

**Authors:** Radek Erban, Hye-Won Kang

**Affiliations:** 1 Mathematical Institute, University of Oxford, Radcliffe Observatory Quarter, Woodstock Road, Oxford, OX2 6GG UK; 2 Department of Mathematics and Statistics, University of Maryland, Baltimore County, 1000 Hilltop Circle, Baltimore, 21250 Maryland USA

**Keywords:** Chemical reaction networks, Limit cycles, Mass action kinetics

## Abstract

The dynamics of a chemical reaction network (CRN) is often modeled under the assumption of mass action kinetics by a system of ordinary differential equations (ODEs) with polynomial right-hand sides that describe the time evolution of concentrations of chemical species involved. Given an arbitrarily large integer $K \in {\mathbb N}$, we show that there exists a CRN such that its ODE model has at least *K* stable limit cycles. Such a CRN can be constructed with reactions of at most second-order provided that the number of chemical species grows linearly with *K*. Bounds on the minimal number of chemical species and the minimal number of chemical reactions are presented for CRNs with *K* stable limit cycles and at most second order or seventh-order kinetics. We also show that CRNs with only two chemical species can have *K* stable limit cycles, when the order of chemical reactions grows linearly with *K*.

## Introduction

Chemical reaction networks (CRNs) are often modeled by reaction rate equations, which are systems of first-order, autonomous, ordinary differential equations (ODEs) describing the time evolution of the concentrations of chemical species involved. Considering CRNs which are subject to the law of mass action, their reaction rate equations have polynomials on their right-hand sides (Yu and Craciun 2018; Craciun et al. 2020). The mathematical investigation of ODEs with polynomial right-hand sides has a long history and includes a number of challenging open mathematical problems, for example, Hilbert’s 16$^{\textrm{th}}$ Problem (Ilyashenko 2002), which asks questions about the number and position of limit cycles of the planar ODE system of the form

1 $$\begin{aligned} \frac{\text{ d }x}{\text{ d }t}= & {} f(x,y), \end{aligned}$$

2 $$\begin{aligned} \frac{\text{ d }y}{\text{ d }t}= & {} g(x,y), \end{aligned}$$

where *f*(*x*, *y*) and *g*(*x*, *y*) are real polynomials of degree at most *n*. Denoting *H*(*n*) the maximum number of limit cycles for the system (1–2), neither the value of *H*(*n*) (for $n \ge 2$) nor any upper bound on *H*(*n*) has yet been found (Ilyashenko 1991). Since a quadratic system with 4 limit cycles has been constructed (Shi 1980), we know that $H(2) \ge 4.$ Similarly, $H(3) \ge 13$, because cubic systems with at least 13 limit cycles have been found (Li et al. 2009; Yang et al. 2010).

Considering CRNs with two chemical species undergoing chemical reactions of at most *n*-th order, their reaction rate equations are given in the form (1–2), where *f*(*x*, *y*) and *g*(*x*, *y*) are real polynomials of degree at most *n*. In particular, if we denote by *C*(*n*) the maximum number of stable limit cycles in such reaction rate equations, then we have $C(n) \le H(n).$ Considering CRNs with two chemical species undergoing chemical reactions of at most second order, it has been previously shown (Póta 1983; Schuman and Tóth 2003) that their reaction rate equations cannot have any limit cycles (i.e., $C(2)=0$), while general ODEs with quadratic right-hand sides can have multiple limit cycles, with $H(2) \ge 4$. In particular, we observe that finding CRNs with two chemical species which have, under mass action kinetics, multiple stable limit cycles, is even more challenging than finding planar polynomial ODEs with multiple limit cycles. Considering cubic systems, we have $H(3) \ge 13$, but most of the chemical systems (with at most third-order reactions) in the literature often have at most one limit cycle (Field and Noyes 1974; Schnakenberg 1979; Plesa et al. 2016). A chemical system with two stable limit cycles has been constructed (Plesa et al. 2017), giving $C(3) \ge 2$, but this is still much less than 13 limit cycles which can be found in some ODE systems with cubic right-hand sides in the literature (Li et al. 2009; Yang et al. 2010). To obtain multiple stable limit cycles in chemical systems, we have to consider higher-order chemical reactions or systems with more than two chemical species (Boros and Hofbauer 2021, 2022).

Considering CRNs with N chemical species undergoing chemical reactions of at most *n*-th order, their reaction rate equations are given as the following system of ODEs

3 $$\begin{aligned} \frac{\text{ d }{{\textbf{x}}}}{\text{ d }t} = {{\textbf{f}}}({{\textbf{x}}}), \end{aligned}$$

where ${{\textbf{x}}} = (x_1,x_2,\dots ,x_N) \in {{\mathbb {R}}}^N$ is the vector of concentrations of *N* chemical species and its right-hand side ${{\textbf{f}}}: {{\mathbb {R}}}^N \rightarrow {{\mathbb {R}}}^N$ is a vector of real polynomials of degree at most *n*. In this paper, we prove the following first main result:

### Theorem 1

Let *K* be an arbitrary positive integer. Then there exists a CRN with *N*(*K*) chemical species which are subject to *M*(*K*) chemical reactions of at most seventh order such that

(i) Reaction rate equations (3) have at least *K* stable limit cycles,

(ii) We have $N(K) \le K+2$ and $M(K) \le 29 \, K$.

Theorem 1 provides a stronger result than finding *K* limit cycles in a polynomial ODE system of the form (3), because not every polynomial ODE system corresponds to a CRN and, therefore, the set of reaction rate equations is a proper subset of ODEs with polynomial right-hand sides. To make the existence of *K* limit cycles possible while restricting to polynomials of degree at most $n=7$, we allow for more than two chemical species, replacing the ODE system (1–2) by a more general ODE system (3) with *N*(*K*) equations. In particular, the next important question is how small the CRN can be so that it has *K* limit cycles. Our answer is partially given in part (ii) of Theorem 1 where we provide upper bounds on the number of chemical species involved and the number of chemical reactions (of at most seventh order). Another important parameter to consider is the maximum order of the chemical reactions involved, i.e., the degree *n* of the polynomials on the right-hand side of the ODE system (3). Since systems of at most second-order reactions (the case $n=2$) are of special interest in the theory of CRNs and applications (Wilhelm 2000), we state our second main result as:

### Theorem 2

Let *K* be an arbitrary positive integer. Then there exists a CRN with *N*(*K*) chemical species which are subject to *M*(*K*) chemical reactions of at most second order such that

(i) Reaction rate equations (3) have at least *K* stable limit cycles,

(ii) We have $N(K) \le 7K+14$ and $M(K) \le 42 \, K + 24$.

By restricting to second-order (bimolecular) reactions, we obtain CRNs with more realistic second-order kinetics, but our construction increases the number of species and chemical reactions involved, as it can be seen by comparing parts (ii) of Theorems 1 and 2. The precise definitions of CRNs, mass action kinetics, reaction rate equations and limit cycles in *N*-dimensional systems are given in Sect. 2.

In both Theorems 1 and 2, we restrict our considerations to systems described by polynomial ODEs where the degree of polynomials is bounded by a constant independent of *K*, i.e., we consider polynomials of the degree at most $n=7$ (in Theorem 1) or at most $n=2$ (in Theorem 2), and we increase the number of chemical species, *N*(*K*), as *K* increases, to get *K* stable limit cycles. Another approach is to restrict our considerations to chemical systems with only $N=2$ chemical species. In Sect. 8, we construct two-species CRNs with *K* stable limit cycles which include chemical reactions of at most *n*(*K*)-th order, where $n(K)=6K-2$. This establishes our third main result:

### Theorem 3

Let *C*(*n*) be the maximum number of stable limit cycles of reaction rate equations (1–2) corresponding to CRNs with two chemical species undergoing chemical reactions of at most *n*-th order. Then we have

4 $$\begin{aligned} C(n) \ge \left\lfloor \frac{n+2}{6} \right\rfloor , \end{aligned}$$

where the floor function $\lfloor . \rfloor$ denotes the integer part of a positive real number.

To prove Theorems 1, 2 and 3, we first construct a planar system given by Eqs. (1–2), where *f* and *g* are continuous non-polynomial functions chosen in such a way that the ODE system (1–2) has *K* stable limit cycles in the positive quadrant $[0,\infty ) \times [0,\infty )$. Such a planar non-polynomial ODE system is constructed in Sect. 3. In Sect. 4, we then increase the number of chemical species from 2 to *N*(*K*) to transform the non-polynomial ODE system to a polynomial one. In Sect. 5, we modify this construction by using an *x*-factorable transformation to arrive at reaction rate equations corresponding to a CRN (Samardzija et al. 1989). Theorem 1 is then proven in Sect. 6 by showing that the reaction rate equations have at least *K* stable limit cycles. This is followed by our proofs of Theorems 2 and 3 in Sects. 7 and 8, respectively.

## Notation and Mathematical Terminology

### Definition 1

A *chemical reaction network* (CRN) is defined as a collection $({\mathcal {S}},{\mathcal {C}},{\mathcal {R}})$ consisting of chemical species ${\mathcal {S}}$, reaction complexes ${\mathcal {C}}$ and chemical reactions ${\mathcal {R}}$. We denote by *N* the number of chemical species and by *M* the number of chemical reactions, i.e., $\vert {\mathcal {S}}\vert =N$ and $\vert {\mathcal {R}}\vert =M$. Each chemical reaction is of the form

5 $$\begin{aligned} \sum _{i=1}^N \nu _{i,j} X_i \;\, \longrightarrow \;\, \sum _{i=1}^N \nu '_{i,j} X_i, \qquad \text{ for } \; j=1,2,\dots ,M, \end{aligned}$$

where $X_i,$ $i=1,2,\dots ,N$, are chemical species, and linear combinations $\sum _{i=1}^N \nu _{i,j} X_i$ and $\sum _{i=1}^N \nu '_{i,j} X_i$ of species with non-negative integers $\nu _{i,j}$ and $\nu '_{i,j}$ are reaction complexes.

### Definition 2

Let $({\mathcal {S}},{\mathcal {C}},{\mathcal {R}})$ be a CRN with *N* chemical species and *M* chemical reactions. Let $x_i(t)$ be the concentration of chemical species $X_i \in {\mathcal {S}}$, $i=1,2,\dots ,N$. The time evolution of $x_i(t)$ is, under the assumption of the mass action kinetics, described by the *reaction rate equations*, which are written as a system of *N* ODEs in the form

6 $$\begin{aligned} \frac{\text{ d }x_i}{\text{ d }t}(t) = \sum _{j=1}^M k_j \, (\nu '_{i,j} - \nu _{i,j}) \, \prod _{\ell =1}^N x_\ell ^{\nu _{\ell ,j}}, \qquad \text{ for } \quad i=1,2,\dots ,N, \end{aligned}$$

where $k_j$, $j=1,2,\dots ,M$, is a positive constant called the reaction rate for the *j*-th reaction.

To illustrate Definitions 1 and 2, we present a simple example system, which is known to have a stable limit cycle for certain parameter values (Schnakenberg 1979; Erban and Chapman 2020).

### Example

Consider a chemical reaction network with two chemical species $X_1$ and $X_2$ which are subject to the following four chemical reactions

7 $$\begin{aligned} 2 X_1 + X_2 {\mathop {\longrightarrow }\limits ^{k_1}}3 X_1,\;\quad \emptyset {\mathop {\longrightarrow }\limits ^{k_2}}X_1,\;\quad X_1 {\mathop {\longrightarrow }\limits ^{k_3}}\emptyset ,\;\quad \emptyset {\mathop {\longrightarrow }\limits ^{k_4}}X_2. \end{aligned}$$

Then $N=\vert {\mathcal {S}}\vert =2$, $M=\vert {\mathcal {R}}\vert =4$ and the set of reaction complexes is given as ${\mathcal {C}}=\{2X_1+X_2,\, 3 X_1,\,X_1,\,X_2,\,\emptyset \}$. Denote $\nu$ and $\nu '$ as matrices with elements $\nu _{i,j}$ and $\nu '_{i,j}$. Then, both are $N \times M = 2\times 4$ matrices given by

$$\begin{aligned} \nu = \begin{bmatrix} 2 &{} 0 &{} 1 &{} 0 \\ 1 &{} 0 &{} 0 &{} 0 \\ \end{bmatrix},\qquad \nu ' = \begin{bmatrix} 3 &{} 1 &{} 0 &{} 0 \\ 0 &{} 0 &{} 0 &{} 1 \\ \end{bmatrix}. \end{aligned}$$

The reaction rate equations (6) are given as the following system of two ODEs

$$\begin{aligned} \frac{\text{ d }x_1}{\text{ d }t}(t)= & {} k_1 x_1^2 x_2 + k_2 - k_3 x_1,\\ \frac{\text{ d }x_2}{\text{ d }t}(t)= & {} - k_1 x_1^2 x_2 + k_4, \end{aligned}$$

which describes the time evolution of the concentrations $x_1(t)$ and $x_2(t)$ of chemical species $X_1$ and $X_2$, respectively.

In general, the reaction rate equations (6) in Definition 2 are ODEs of the form (3), where the right-hand side ${{\textbf{f}}}: {{\mathbb {R}}}^N \rightarrow {{\mathbb {R}}}^N$ is a vector of real polynomials. However, not every polynomial ODE system can be obtained as the reaction rate equations of a CRN as we formalize in Lemma 1, where we provide a necessary and sufficient condition (9) when a polynomial ODE system can be written as reaction rate equations of a CRN. The condition is that any polynomial right-hand side terms not proportional to $x_i$ in the equation for chemical species $X_i$ are non-negative. The necessity of the condition (9) is shown based on the fact that any reaction rate equations of a CRN satisfy this condition. The sufficiency of the condition is provided by showing that each term satisfying the condition in a polynomial ODE system can correspond to a chemical reaction.

### Lemma 1

Consider a system of *N* ODEs with polynomial right-hand sides describing the time evolution of $x_i(t)$, $i=1,2,\dots ,N,$ in the form

8 $$\begin{aligned} \frac{\text{ d }x_i}{\text{ d }t}(t) = \sum _{j=1}^M \alpha _{i,j} \, \prod _{\ell =1}^N x_\ell ^{\nu _{\ell ,j}}, \qquad \text{ for } \quad i=1,2,\dots ,N, \end{aligned}$$

where $\alpha _{i,j}$ are real constants and $\nu _{i,j}$ are non-negative integers, for $i=1,2,\dots ,N$ and $j=1,2,\dots ,M.$ Then the polynomial ODE system (8) can be written as reaction rate equations (6) of a CRN if and only if

9 $$\begin{aligned} \nu _{i,j} = 0 \quad \text{ implies } \quad \alpha _{i,j} \ge 0 \quad \hbox {for any}\; i=1,2,\dots ,N \;\hbox {and}\; j=1,2,\dots ,M. \end{aligned}$$

### Proof

Reaction rate equations (6) are of the form (8). The non-negativity condition (9) follows from $\nu _{i,j}=0$ and the non-negativity of both $k_j$ and $\nu '_{i,j}$ in Eq. (6).

Conversely, if an ODE is of the form (8) and $\alpha _{i,j}>0$, then we can choose $\nu '_{i,j}=\nu _{i,j}+1$ in Eq. (6) and put the reaction rate as $k_j = \alpha _{i,j}$. On the other hand, if $\alpha _{i,j} < 0$, then the condition (9) implies that $\nu _{i,j} \ge 1$, because $\nu _{i,j}$ are non-negative integers. Therefore, we can put $\nu '_{i,j}=\nu _{i,j}-1$ and $k_j = -\alpha _{i,j}>0.$ $\square$

In this paper, we prove the existence of limit cycles in chemical systems in Sects. 6, 7 and 8 by proving the existence of limit cycles in systems of ODEs (8) with polynomial right-hand sides satisfying the condition (9). Then the approach used in the proof of Lemma 1 can be used to construct the corresponding CRN. However, the construction of a CRN corresponding to reaction rate equations is not unique. For example, consider a term of the form $-x_1^3$ on the right-hand side of Eq. (8). Using the construction in the proof of Lemma 1, it would correspond to the chemical reaction $3X \longrightarrow 2X$ with the rate constant equal to 1, but the same term can also correspond to the chemical reaction $3X \longrightarrow X$ with the rate constant equal to 1/2. We conclude this section by a formal definition of a stable limit cycle.

### Definition 3

Consider a system of *N* ODEs given by (3), where their right-hand side ${{\textbf{f}}}: {{\mathbb {R}}}^N \rightarrow {{\mathbb {R}}}^N$ is continuous. A stable *limit cycle* is a trajectory ${\textbf{x}}_{c}(t)$ for $t \in [0,\infty )$ such that

(i) ${\textbf{x}}_{c}(t)$ is a solution of the ODE system (3),
(ii) There exists a positive constant $T>0$ such that ${\textbf{x}}_{c}(T)={\textbf{x}}_{c}(0)$ and ${\textbf{x}}_{c}(t) \ne {\textbf{x}}_{c}(0)$ for $0< t < T,$
(iii) There exists $\varepsilon >0$ such that

$$\text{ dist }\{{\textbf{x}}(0),{\textbf{x}}_{c}\} <\varepsilon $$ implies $$\text{ dist }\{{\textbf{x}}(t),{\textbf{x}}_{c}\} \rightarrow 0$$ as $$t \rightarrow \infty .$$

In Definition 3, the constant *T* is the period of the limit cycle and the property (iii) states that the limit cycle attracts all trajectories which start sufficiently close to it. The distance in the property (iii) of Definition 3 is the Euclidean distance defined by

$$\begin{aligned} \text{ dist }\{{\textbf{z}},{\textbf{x}}_{c}\} = \min _{t \in [0,T]} \text{ dist }\{{\textbf{z}},{\textbf{x}}_{c}(t)\} = \min _{t \in [0,T]} \left( \sum _{i=1}^N \left( z_i - x_{c,i}(t) \right) ^2 \right) ^{1/2} \end{aligned}$$

for $${\textbf{z}} = [z_1,z_2,\dots ,z_N] \in {{\mathbb {R}}^N}$$ and $${\textbf{x}}_{c}(t) = [x_{c,1}(t),x_{c,2}(t),\dots ,x_{c,N}(t)] \in {{\mathbb {R}}^N}$$.

## Planar ODE Systems with Arbitrary Number of Limit Cycles

In this section, we construct a planar ODE system of the form (1–2) with *K* limit cycles in the positive quadrant. It is constructed as a function of 2*K* parameters denoted by $a_1,$ $a_2,$ $\dots ,$ $a_K$ and $b_1,$ $b_2,$ $\dots ,$ $b_K,$ as

10 $$\begin{aligned} \frac{\text{ d }x}{\text{ d }t} \!= & {} \! \sum _{k=1}^K \frac{(x-a_k) \big \{1-(x-a_k)^2-(y-b_k)^2\big \} -(y-b_k)}{ 1 + (x-a_k)^6 + (y-b_k)^6} \,=f(x,y), \quad \; \end{aligned}$$

11 $$\begin{aligned} \frac{\text{ d }y}{\text{ d }t} \!= & {} \! \sum _{k=1}^K \frac{(y-b_k) \big \{1-(x-a_k)^2-(y-b_k)^2\big \} +(x-a_k)}{ 1 + (x-a_k)^6 + (y-b_k)^6} \,=g(x,y). \quad \; \end{aligned}$$

**Fig. 1:**
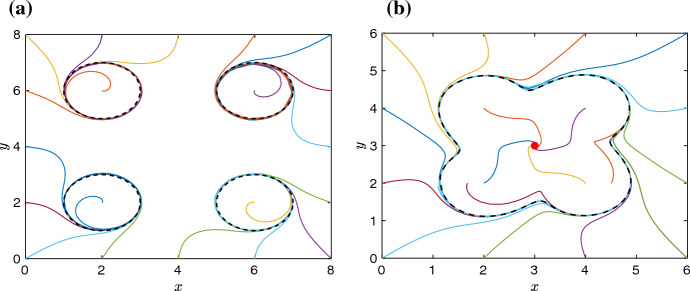
**a** Twenty illustrative trajectories of the ODE system (10–11) for $K=4$ and the parameter choices $a_1 = b_1 = a_2 = b_3 = 2$ and $a_3 = b_2 = a_4 = b_4 = 6$. As $t \rightarrow \infty$, all presented trajectories approach one of the four limit cycles, which are plotted as the black dashed lines. **b** Twenty illustrative trajectories of the ODE system (10–11) for $K=4$ and the parameter choices $a_1 = b_1 = a_2 = b_3 = 2$ and $a_3 = b_2 = a_4 = b_4 = 4$. As $t \rightarrow \infty$, some trajectories converge to the stable limit cycle denoted by the black dashed line, while some trajectories, which started inside the limit cycle, converge to the stable fixed point denoted as the red dot (Color figure online)

An illustrative dynamics of the ODE system (10–11) is shown in Fig. 1(a) for $K=4$,

where the ODE system has four limit cycles, which is highlighted in Fig. 1a by plotting some representative trajectories. The existence of *K* stable limit cycles for the ODE system (10–11) can also be proven analytically, as it is done in Lemma 2.

### Remark

To construct the ODE system (10–11), one can first consider a planar ODE system written as $\text{ d }r/\text{d }t = r(1-r^2)$ in the polar coordinate system. It has one stable limit cycle (a unit circle) and can be rewritten in the Cartesian coordinate system as

12 $$\begin{aligned} \frac{\text{ d }x}{\text{ d }t}= & {} x(1-x^2-y^2)-y, \end{aligned}$$

13 $$\begin{aligned} \frac{\text{ d }y}{\text{ d }t}= & {} y(1-x^2-y^2)+x. \end{aligned}$$

To obtain the ODE system (10–11), we translate the right-hand sides of ODEs (12–13) by $(x,y)\rightarrow (x-a_k,y-b_k)$ so that the corresponding limit cycle is centered at the point $(a_k,b_k)$. Then we add the *K* terms (corresponding to *K* limit cycles) together with suitable weights. Note that the denominator of the *k*-th term in (10–11) is approximately one when (*x*, *y*) is close to $(a_k,b_k)$, while the *k*-th term in (10–11) is approximately zero when (*x*, *y*) is far away from the point $(a_k,b_k)$.

In Fig. 1a, we have presented an example with $K=4$ and parameter choices

$$\begin{aligned} (a_1,b_1) = (2,2), \quad (a_2,b_2) = (2,6), \quad (a_3,b_3) = (6,2) \quad \text{ and } \quad (a_4,b_4) = (6,6). \end{aligned}$$

In particular, the distance between points $(a_i,b_i)$, $i=1,2,3,4,$ is at least four. If we decrease this distance, then the ODE system (10–11) can have less limit cycles. This is highlighted in Fig. 1b, where we present an example with $K=4$ and parameter choices

$$\begin{aligned} (a_1,b_1) = (2,2), \quad (a_2,b_2) = (2,4), \quad (a_3,b_3) = (4,2) \quad \text{ and } \quad (a_4,b_4) = (4,4). \end{aligned}$$

In Fig. 1b, we observe that there is only one limit cycle, denoted as the black dashed line. This limit cycle is stable and a number of illustrative trajectories converge to this limit cycle as $t \rightarrow \infty$. However, there is also a stable equilibrium point at (3, 3), which attracts some of the trajectories. In particular, we can only expect that the ODE system (10–11) will have *K* stable limit cycles provided that points $(a_i,b_i)$ are sufficiently separated. This is formally proven in Lemma 2, by defining *K* disjoint open sets (15), where each is an annulus with a center $(a_i,b_i)$ for $i=1,2,\dots ,K$. First, we show that each annulus does not contain any equilibrium points. Then, we show that a directional vector of the ODE system (10–11) on the boundary of each annulus points inside the domain. Applying the Poincaré-Bendixson theorem (Strogatz 2015), we conclude that each annulus contains at least one stable limit cycle; thus, the ODE system (10–11) has at least *K* stable limit cycles.

### Lemma 2

Let us assume that

14 $$\begin{aligned} (a_i-a_j)^2+(b_i-b_j)^2 > 15\left( K^{2/3}+2\right) \qquad \text{ for } \text{ all } \quad i \ne j, \end{aligned}$$

where $i,j=1,2,\dots ,K.$ Then the ODE system (10–11) has at least *K* stable limit cycles.

### Proof

We define the sets

15 $$\begin{aligned} \Omega _i = \left\{ (x,y) \,: \, 1/2< (x-a_i)^2 + (y-b_i)^2 < 2 \right\} , \quad \; \text{ for } \;\; i=1,2,\dots ,K. \quad \end{aligned}$$

Then the condition (14) implies that

$$\begin{aligned} \Omega _i \cap \Omega _j = \emptyset , \qquad \text{ for } \text{ all } \quad i \ne j, \quad \text{ where } \quad i,j=1,2,\dots ,K, \end{aligned}$$

i.e., the sets $\Omega _i$ are pairwise disjoint sets. We will show that each of them contains at least one stable limit cycle. The boundary of $\Omega$ consists of two parts: outer and inner circles defined by

16 $$\begin{aligned} \partial \Omega _{i1} = \left\{ (x,y): (x - a_i)^2 + (y - b_i)^2 = 2 \right\} \end{aligned}$$

and

17 $$\begin{aligned} \partial \Omega _{i2} = \left\{ (x,y): (x - a_i)^2 + (y - b_i)^2 = 1/2 \right\} , \end{aligned}$$

respectively, that is, $\partial \Omega _i =\partial \Omega _{i1} \cup \partial \Omega _{i2}$. Define the following functions for $k=1,2,\dots ,K$:

18 $$\begin{aligned} f_k(z_1,z_2)= & {} \frac{ z_1 \big \{ 1-z_1^2-z_2^2 \big \} -z_2}{ 1 + z_1^6 + z_2^6 }, \end{aligned}$$

19 $$\begin{aligned} g_k(z_1,z_2)= & {} \frac{z_2 \big \{1-z_1^2-z_2^2\big \} +z_1}{ 1 + z_1^6 + z_2^6}. \end{aligned}$$

Then, the ODE system (10–11) can be rewritten as

20 $$\begin{aligned} \frac{\text{ d }x}{\text{ d }t} \!= & {} \! f(x,y) , \quad \; \end{aligned}$$

21 $$\begin{aligned} \frac{\text{ d }y}{\text{ d }t} \!= & {} \! g(x,y) , \quad \; \end{aligned}$$

where

22 $$\begin{aligned} f(x,y) = \sum _{k=1}^K f_k(x-a_k,y-b_k) \quad \text{ and } \quad g(x,y) = \sum _{k=1}^K g_k(x-a_k,y-b_k). \end{aligned}$$

First, we will show that $\Omega _i$ for $i=1,2,\dots , K$ does not contain any equilibrium points. Let us consider any point $(x^*,y^*)\in \Omega _i$. Substituting

23 $$\begin{aligned} x^*=a_i+r\cos {\theta }, \quad y^*=b_i+r\sin {\theta }, \end{aligned}$$

in the terms for $k=i$ in (22), we obtain

24 $$\begin{aligned} f(x^*,y^*) \!= & {} \! \frac{r\cos {\theta } \, \{1-r^2\}-r\sin {\theta }}{1+r^6\cos ^6{\theta }+r^6\sin ^6{\theta }} \, + \sum _{k=1,k\ne i}^K f_k(x^*-a_k,y^*-b_k) , \quad \; \end{aligned}$$

25 $$\begin{aligned} g(x^*,y^*) \!= & {} \! \frac{r\sin {\theta }\, \{1-r^2\}+r\cos {\theta }}{1+r^6\cos ^6{\theta }+r^6\sin ^6{\theta }} \, + \sum _{k=1,k\ne i}^K g_k(x^*-a_k,y^*-b_k) . \quad \; \end{aligned}$$

The first terms in (24) and (25) can be rewritten as

26 $$\begin{aligned} \frac{4 r\sqrt{(r^2-1)^2+1}\,\, \sin (\theta +{\tilde{\theta }})}{4 +r^6\left( 4- 3 \sin ^2{2\theta }\right) } \,, \end{aligned}$$

where ${\tilde{\theta }}=\alpha$ with $\tan {\alpha }=r^2-1$ and $\pi /2< \alpha < 3\pi /2$ in the case of (24) and ${\tilde{\theta }}=\alpha -\pi /2$ in the case of (25). Since we have

$$\begin{aligned} \max ( \vert \sin (\theta +\alpha )\vert , \vert \sin (\theta +\alpha -\pi /2)\vert ) > 1/\sqrt{2} \end{aligned}$$

for any $\theta$ and $\alpha$, at least one of the two terms expressed in the form (26) is greater than

$$\begin{aligned} \frac{1}{\sqrt{2}}\frac{r\sqrt{(r^2-1)^2+1}}{1+r^6} \,, \end{aligned}$$

which has a minimum $\sqrt{2}/9$ when $1/2<r^2<2$. Therefore, at least one of the absolute values of the *i*-th components, $f_i(x^*-a_i,y^*-b_i)$ and $g_i(x^*-a_i,y^*-b_i)$, in (24) and (25) at any point $(x^*,y^*)\in \Omega _i$ is greater than or equal to $\sqrt{2}/9$. Without loss of generality, we assume

$$\begin{aligned} \vert f_i(x^*-a_i,y^*-b_i) \vert \ge \vert g_i(x^*-a_i,y^*-b_i) \vert . \end{aligned}$$

Then we have $\vert f_i(x^*-a_i,y^*-b_i) \vert \ge \sqrt{2}/9$. We want to show that the second term in (24) (i.e., the sum) has a smaller magnitude than the first term $f_i(x^*-a_i,y^*-b_i)$ so that we could conclude that $f(x^*,y^*)\ne 0$. The *k*-th component in the second term in (24) is bounded by

27 $$\begin{aligned} \left| f_k(z_1,z_2)\right|\le & {} \frac{\vert z_1\vert \, \vert 1-z_1^2-z_2^2\vert +\vert z_2\vert }{\vert 1+z_1^6+z_2^6\vert } \end{aligned}$$

where $(z_1,z_2)=(x^*-a_k,y^*-b_k)$. Denoting $c^2 = z_1^2+z_2^2$, we have

28 $$\begin{aligned} 1+\frac{c^6}{4}\le 1+z_1^6+z_2^6\le 1+c^6. \end{aligned}$$

Using $\vert z_i\vert \le c$ and (28), we estimate (27) as

29 $$\begin{aligned} \left| f_k(z_1,z_2)\right| \le \frac{c \left( |1-c^2|+1\right) }{1+c^6/4 } \,. \end{aligned}$$

Since $(x^*,y^*)\in \Omega _i$ and $(a_k,b_k)\in \Omega _k$ where $k\ne i$, our assumption (14) implies that $c^2\ge 2$. Thus, (29) becomes

30 $$\begin{aligned} \left| f_k(z_1,z_2)\right| \le \frac{c^3}{1+c^6/4} \le \frac{4}{c^3} \,. \end{aligned}$$

Therefore, the magnitude of the second term in (24) is bounded by $4(K-1)/c^3$. Since $\vert f_i(x^*-a_i,y^*-b_i) \vert \ge \sqrt{2}/9$, a sufficient condition for $f(x^*,y^*)\ne 0$ is

31 $$\begin{aligned} \frac{4(K-1)}{c^3} < \frac{\sqrt{2}}{9} \,. \end{aligned}$$

Using the assumption (14), the distance $c=\sqrt{(x^*-a_k)^2+(y^*-b_k)^2}$ is bounded by

32 $$\begin{aligned} c> \sqrt{(a_i-a_k)^2+(b_i-b_k)^2}-\sqrt{2} > \sqrt{15\left( K^{2/3}+2\right) } -\sqrt{2} \,, \end{aligned}$$

which implies the sufficient condition (31). Therefore, $(x^*,y^*)$ is not an equilibrium point.

Next, consider an arbitrary point $(x_b,y_b) \in \partial \Omega _{i1}$. Let us calculate the scalar product of vectors

33 $$\begin{aligned} (x_b-a_i,y_b-b_i) \quad \text{ and } \quad \big (f(x_b,y_b), g(x_b,y_b)\big ) \,. \end{aligned}$$

Using (22), we obtain this scalar product as

34 $$\begin{aligned}{} & {} (x_b - a_i) f_i(x_b-a_i,y_b-b_i) + (y_b - b_i) g_i(x_b-a_i,y_b-b_i) \nonumber \\{} & {} + (x_b - a_i) \!\!\!\! \sum _{k=1,k \ne i}^K f_k(x_b-a_k,y_b-b_k)+ (y_b - b_i) \!\!\!\! \sum _{k=1, k \ne i}^K g_k(x_b-a_k,y_b-b_k).\nonumber \\ \end{aligned}$$

The first two terms in (34) become

$$\begin{aligned} \frac{-2}{1+(x_b-a_i)^6+(y_b-a_i)^6} \,, \end{aligned}$$

which has a magnitude greater than 2/9 by using (28) with $c^2=(x_b-a_i)^2+(y_b-b_i)^2=2$. Using (30), $\vert x_b-a_i\vert \le \sqrt{2}$ and $\vert y_b-b_i\vert \le \sqrt{2}$, we can estimate the third and fourth terms in (34), namely, we have

35 $$\begin{aligned} \left| (x_b-a_i) \, f_k(z_1,z_2)\right| \le \frac{4\sqrt{2}}{d^3} \quad \text{ and } \quad \left| (y_b-b_i) \, g_k(z_1,z_2)\right| \le \frac{4\sqrt{2}}{d^3} \,, \end{aligned}$$

where $d^2=(x_b-a_k)^2+(y_b-b_k)^2$. Then the sum of the third and fourth terms in (34) is bounded by $8\sqrt{2}(K-1)/d^3$. Therefore, the sufficient condition that the scalar product in (34) is negative is

36 $$\begin{aligned} \frac{8\sqrt{2}(K-1)}{d^3} < \frac{2}{9}. \end{aligned}$$

Using the assumption (14), the distance $d=\sqrt{(x_b-a_k)^2+(y_b-b_k)^2}$ is bounded by

37 $$\begin{aligned} d> \sqrt{(a_i-a_k)^2+(b_i-b_k)^2}-\sqrt{2} > \sqrt{15\left( K^{2/3}+2\right) } -\sqrt{2} \,, \end{aligned}$$

which implies the sufficient condition (36). Therefore, the vector

$$\begin{aligned} \big (f(x_b,y_b), g(x_b,y_b)\big ) \end{aligned}$$

always points inside the domain $\Omega _i$ for each boundary point $(x_b,y_b) \in \partial \Omega _{i1}$.

Similarly, for an arbitrary point $(x_b,y_b)\in \partial \Omega _{i2}$, we can show that the scalar product of vectors in (33) is always positive due to that the sum of the first two terms in (34) is equal to

$$\begin{aligned} \frac{1/4}{1+(x_b-a_i)^6+(y_b-b_i)^6} \,, \end{aligned}$$

which is greater than 2/9 by using (28) with $c^2=1/2$, and the sum of the third and fourth terms in (34) is bounded by $8(K-1)/(d^3 \sqrt{2})$. Therefore, the sufficient condition that the scalar product in (34) is positive is

$$\begin{aligned} \frac{8(K-1)}{d^3 \sqrt{2}} < \frac{2}{9} \,, \end{aligned}$$

which is a weaker condition than the condition (36), i.e., it is again satisfied because of our assumption (14). This implies that the scalar product in (34) is positive. Thus, the directional vector always points inside the domain $\Omega _i$ on all parts of the boundary $\partial \Omega _i$.

Therefore, applying Poincaré-Bendixson theorem (Strogatz 2015), we conclude that each $\Omega _i$ contains at least one stable limit cycle. Since $\Omega _i$, $i=1,2,\dots ,K,$ are pairwise disjoint, this implies that the ODE system (10–11) has at least *K* stable limit cycles. $\square$

## ODE Systems with Polynomial Right-Hand Sides and Arbitrary Number of Limit Cycles

Considering an auxiliary variable

38 $$\begin{aligned} u_i=\frac{1}{1 + (x-a_i)^6 + (y-b_i)^6}, \; \text{ for } \quad i=1,2,\dots ,K, \end{aligned}$$

we can formally convert the non-polynomial ODE system (10–11) to a system of $(K+2)$ ODEs with polynomial right-hand sides (Kerner 1981). We obtain

39 $$\begin{aligned} \frac{\text{ d }x}{\text{ d }t}&= \sum _{k=1}^K u_k \left[ (x-a_k) \big \{1-(x-a_k)^2-(y-b_k)^2\big \} -(y-b_k)\right] , \end{aligned}$$

40 $$\begin{aligned} \frac{\text{ d }y}{\text{ d }t}&= \sum _{k=1}^K u_k \left[ (y-b_k) \big \{1-(x-a_k)^2-(y-b_k)^2\big \} +(x-a_k)\right] , \end{aligned}$$

41 $$\begin{aligned} \frac{\text{ d }u_i}{\text{ d }t}&= - 6 u_i^2 (x - a_i)^5 \sum _{k=1}^K u_k \left[ (x-a_k) \big \{1-(x-a_k)^2-(y-b_k)^2\big \} -(y-b_k)\right] \nonumber \\&\quad -6 u_i^2 (y - b_i)^5 \sum _{k=1}^K u_k \left[ (y-b_k) \big \{1-(x-a_k)^2-(y-b_k)^2\big \} +(x-a_k)\right] , \end{aligned}$$

for $i=1,2,\dots ,K$. The dynamics of the original ODE system (10–11) with the initial condition $(x(0),y(0))=(x_0,y_0)$ is the same as the dynamics of the extended ODE system (39–41), when we initialize the additional variables by

42 $$\begin{aligned} u_i(0) = \frac{1}{1 + (x_0-a_i)^6 + (y_0-b_i)^6}, \; \text{ for } \quad i=1,2,\dots ,K. \end{aligned}$$

**Fig. 2:**
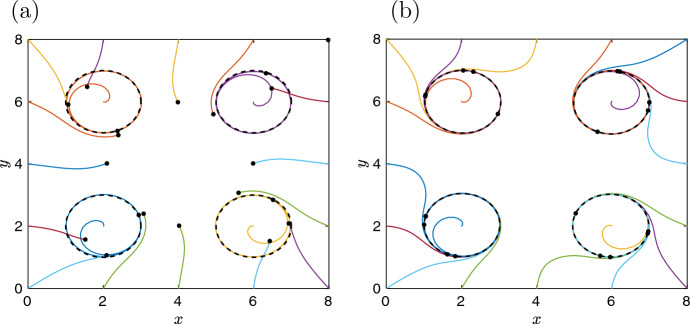
**a** Twenty illustrative trajectories of the ODE system (39–41) for $K=4$, the parameter choices $a_1 = b_1 = a_2 = b_3 = 2$, $a_3 = b_2 = a_4 = b_4 = 6$ and initial condition (43) with $c=1/2$. The black dots denote the final position of each calculated trajectory at time $t=100$. The black dashed lines are limit cycles shown in Fig. 1a. **b** Twenty illustrative trajectories of the ODE system (44–46) for $K=4$, the parameter choices $\varepsilon =1$, $a_1 = b_1 = a_2 = b_3 = 2$, $a_3 = b_2 = a_4 = b_4 = 6$ and the initial condition (43) with $c=1/2$. As $t \rightarrow \infty$, all trajectories approach one of the four limit cycles, which are plotted as the black dashed lines. The black dots denote the final position of each calculated trajectory at time $t=100$ (Color figure online)

However, when we use a general initial condition,

$$\begin{aligned} (x(0),y(0),u_1(0),u_2(0),\dots ,u_K(0)) \in {{\mathbb {R}}}^{K+2}, \end{aligned}$$

the trajectory of the extended ODE system (39–41) may become unbounded and it may not converge to a limit cycle. To illustrate this behavior, let us consider the initial condition

43 $$\begin{aligned} u_i(0) = \frac{c}{1 + (x_0-a_i)^6 + (y_0-b_i)^6}, \; \text{ for } \quad i=1,2,\dots ,K, \end{aligned}$$

where $c>0$ is a constant. If $c=1$, then the initial condition (43) reduces to (42). In particular, Fig. 1a shows an illustrative behavior of both the extended ODE system (39–41) for $c=1$ and the planar ODE system (10–11), assuming that we use a sufficiently accurate numerical method to solve ODEs (39–41) and plot the projection of the calculated trajectory to the (*x*, *y*)-plane. Changing $c=1$ to $c=0.5$, we plot the dynamics of the extended ODE system in Fig. 2a, where the black dots denote the end points of the calculated trajectories at the final time ($t=100$). We observe that only the trajectories which started ‘inside a limit cycle’ (i.e., their projections to the (*x*, *y*)-plane are initially inside a black dashed circle) seem to converge to it, while the other trajectories do not seem to approach the ‘limit cycles’. This is indeed the case even if we continue these trajectories for times $t>100.$ In fact, depending on the accuracy of the numerical method used, all trajectories eventually stop somewhere in the phase plane, because $u_i(t) \rightarrow 0$ as $t \rightarrow \infty$.

On the other hand, considering the extended ODE system (39–41) with the initial condition (43) for $c>1$, some additional variables $u_i(t)$ tend to infinity as $t \rightarrow \infty$, and we again do not observe sustained oscillations in our numerical experiments (results not shown). In particular, the formal conversion of the non-polynomial ODE system (10–11) into the polynomial system (39–41) does not preserve the dynamics well. Therefore, we augment our polynomial ODE system (10–11) in a different way. We introduce *K* new variables $v_i$, $i=1,2,\dots ,K$, and formulate the extended ODE system as the following $(K+2)$ equations:

44 $$\begin{aligned} \frac{\text{ d }x}{\text{ d }t} \!= & {} \! \sum _{k=1}^K v_k \left[ (x-a_k) \big \{1-(x-a_k)^2-(y-b_k)^2\big \} -(y-b_k)\right] , \quad \; \end{aligned}$$

45 $$\begin{aligned} \frac{\text{ d }y}{\text{ d }t} \!= & {} \! \sum _{k=1}^K v_k \left[ (y-b_k) \big \{1-(x-a_k)^2-(y-b_k)^2\big \} +(x-a_k)\right] , \quad \; \end{aligned}$$

46 $$\begin{aligned} \varepsilon \, \frac{\text{ d }v_i}{\text{ d }t} \!= & {} \! 1 - v_i \left[ 1 + (x - a_i)^6 + (y - b_i)^6 \right] , \quad \text{ for } \quad i=1,2,\dots ,K, \end{aligned}$$

where $\varepsilon > 0$ is a constant. The first two ODEs (44–45) are the same as ODEs (39–40) with $v_k$ taking place of $u_k$. The difference is in the dynamics of the additional variables, i.e., in Eq. (46) which removes the non-polynomial factor (38) in a different way. Rather than defining new variable $u_i$ in the form (38) and deriving ODEs which have equivalent dynamics to the ODE system (10–11) for very special initial condition (42), we have written the ODE (46) in such a way that it formally recovers the non-polynomial factor (38) in the limit $\varepsilon \rightarrow 0$, which will be used in our proof of Lemma 3, where we consider small values of $\varepsilon$. However, even for larger values of $\varepsilon$, the ODE system (44–46) has multiple limit cycles for general initial conditions, as it is illustrated for $\varepsilon =1$ and $K=4$ in Fig. 2b, where all plotted trajectories finish on a limit cycle (see the final calculated positions, at time $t=100$, plotted as black dots).

Next, we prove that the extended ODE system (44–46) has *K* limit cycles in the sense of Definition 3 for general values of *K*. Since (44–46) is a system of $(K+2)$ ODEs, we cannot directly apply the Poincaré-Bendixson theorem as we did for the planar system in the proof of Lemma 2. While one possible approach to proving the existence of limit cycles is to work with generalizations of the Poincaré-Bendixson theorem to higher-dimensional ODEs (Hirsch 1982; Li and Muldowney 1996; Sanchez 2010), we will base our proof of Lemma 3 on the application of Tikhonov’s theorem (Tikhonov 1952; Klonowski 1983) and the result of Lemma 2. In particular, we show that the extended system (44–46) is a polynomial system which has *K* limit cycles for sufficiently small values of $\varepsilon$ provided that the points $(a_i,b_i)$ are sufficiently separated, as we have previously established in Lemma 2 for the planar ODE system (10–11).

### Lemma 3

Let us assume that parameters $a_i>0$ and $b_i>0$, $i=1,2,\dots ,K,$ satisfy the inequality (14). Then there exists $\varepsilon _0>0$ such that the ODE system (44–46) has at least *K* stable limit cycles for all $\varepsilon \in (0,\varepsilon _0).$

### Proof

Let us consider $\varepsilon =0$. Then the right-hand side of the ODE (46) is equal to zero. This equation can be solved for $v_i$, $i=1,2,\dots ,K,$ to obtain $v_i = q_i(x,y),$ where we define

47 $$\begin{aligned} q_i(x,y) = \frac{1}{1 + (x - a_i)^6 + (y - b_i)^6}. \end{aligned}$$

Substituting $v_i=q_i(x,y)$ into (44–45), we obtain that the reduced problem in the sense of Tikhonov’s theorem (Tikhonov 1952; Klonowski 1983) is equal to

48 $$\begin{aligned} \frac{\text{ d }{\overline{x}}}{\text{ d }t}= & {} f({\overline{x}},{\overline{y}}), \quad \; \end{aligned}$$

49 $$\begin{aligned} \frac{\text{ d }{\overline{y}}}{\text{ d }t}= & {} g({\overline{x}},{\overline{y}}), \quad \; \end{aligned}$$

where functions $f(\cdot ,\cdot )$ and $g(\cdot ,\cdot )$ are defined in (10) and (11). This means that the reduced system (48–49) corresponding to the fast–slow extended ODE system (44–46) is the same as our original non-polynomial ODE system (10–11). Therefore, using Lemma 2, we know that the reduced system (48–49) has (at least) *K* stable limit cycles in the sense of Definition 3, i.e., there exist *K* solutions

50 $$\begin{aligned} ({\overline{x}}_{c,i}(t), {\overline{y}}_{c,i}(t)) \qquad \text{ for } \quad t \in [0,\infty ), \quad i = 1,2,\dots ,K, \end{aligned}$$

of the reduced system (48–49) which are periodic with period $T_i>0$ for $i=1,2,\dots ,K$. Moreover, there exist $\varepsilon _i>0$, $i=1,2,\dots ,K$, such that any solution $({\overline{x}}(t),{\overline{y}}(t))$ of the reduced systems (48–49) approaches the limit cycle $({\overline{x}}_{c,i}(t), {\overline{y}}_{c,i}(t))$ as $t \rightarrow \infty$ provided that the initial condition $({\overline{x}}(0),{\overline{y}}(0))$ satisfies

51 $$\begin{aligned} \min _{t \in [0,T_i]} \big ({\overline{x}}(0)-{\overline{x}}_{c,i}(t)\big )^2 + \big ({\overline{y}}(0)-{\overline{y}}_{c,i}(t)\big )^2 <\varepsilon _i. \end{aligned}$$

Next, we define pairwise disjoint sets $\Omega _i \subset {\mathbb {R}}^{K+2}$ for $i=1,2,\dots ,K$ by

52 $$\begin{aligned} \Omega _i= & {} \bigg \{ (x,y,v_1,v_2,\dots ,v_K) \in {\mathbb {R}}^{K+2} \quad \text{ such } \text{ that } \nonumber \\{} & {} \min _{t \in [0,T_i]} \big (x-{\overline{x}}_{c,i}(t)\big )^2 + \big (y-{\overline{y}}_{c,i}(t)\big )^2 + \sum _{j=1}^K \big (v_j - q_j({\overline{x}}_{c,i}(t),{\overline{y}}_{c,i}(t))\big )^2 <\varepsilon _i \bigg \},\nonumber \\ \end{aligned}$$

where functions $q_j(\cdot ,\cdot )$ are defined by (47). Let us define

$$\begin{aligned} \varepsilon _0 = \min _{i \in \{1, 2, \dots , K \} } \varepsilon _i. \end{aligned}$$

Let $\varepsilon \in (0,\varepsilon _0)$ be chosen arbitrarily. To show that the extended fast–slow polynomial ODE system (44–46) has (at least) *K* stable limit cycles, it is sufficient to show that each set $\Omega _i$ contains one stable limit cycle. We will do this by applying Tikhonov’s theorem (Tikhonov 1952; Klonowski 1983). Considering the ODEs (46) for $i=1,2,\dots ,K,$ where $x>0$ and $y>0$ are taken as parameters, we obtain the adjoined system as a *K*-dimensional system of ODEs with an isolated stable equilibrium point $[q_1(x,y),q_2(x,y),\dots ,q_K(x,y)]$, where $q_i(\cdot ,\cdot )$ is defined in (47). This equilibrium point attracts the solutions of the adjoined system for any initial condition. Therefore, the ODE system (44–46) has a limit cycle in $\Omega _i$. Moreover, this limit cycle attracts any solution $\big (x(t),y(t),v_1(t),v_2(t),\dots ,v_K(t)\big )$ of the ODE system (44–46) with the initial condition satisfying $\big (x(0),y(0),v_1(0),v_2(0),\dots ,v_K(0)\big ) \in \Omega _i.$ $\square$

## Chemical Systems with Arbitrary Many Limit Cycles

To construct a CRN with *K* limit cycles, we first construct a system of ODEs with polynomial right-hand sides which satisfy the condition (9) in Lemma 1, i.e., it will be a system of reaction rate equations, which correspond to a CRN. Once we have such reaction rate equations, there are infinitely many CRNs described by them, so we conclude this section by specifying some illustrative CRNs corresponding to the derived reaction rate equations.

Our starting point is the polynomial ODE system (44–46), which has *K* limit cycles provided that the conditions of Lemma 3 are satisfied. The reaction rate equations are constructed by applying the so-called *x*-factorable transformation (Plesa et al. 2016) to the right-hand sides of equations (44) and (45). We do not modify the right-hand sides of ODEs (46), because they already satisfy the conditions of Definition 2. We obtain the ODE system:

53 $$\begin{aligned} \frac{\text{ d }x}{\text{ d }t} \!= & {} \! \sum _{k=1}^K x \, v_k \left[ (x-a_k) \big \{1-(x-a_k)^2-(y-b_k)^2\big \} -(y-b_k)\right] , \quad \; \end{aligned}$$

54 $$\begin{aligned} \frac{\text{ d }y}{\text{ d }t} \!= & {} \! \sum _{k=1}^K y \, v_k \left[ (y-b_k) \big \{1-(x-a_k)^2-(y-b_k)^2\big \} +(x-a_k)\right] , \quad \; \end{aligned}$$

55 $$\begin{aligned} \varepsilon \, \frac{\text{ d }v_i}{\text{ d }t} \!= & {} \! 1 - v_i \left[ 1 + (x - a_i)^6 + (y - b_i)^6 \right] , \qquad \text{ for } \quad i=1,2,\dots ,K. \end{aligned}$$

**Fig. 3:**
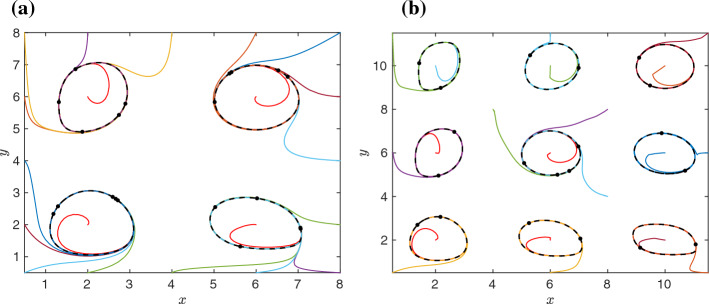
**a** Twenty illustrative trajectories of the ODE system (53–55) for $K=4$, the parameter choices $a_1 = b_1 = a_2 = b_3 = 2$, $a_3 = b_2 = a_4 = b_4 = 6$, $\varepsilon =1$ and the initial condition (43) with $c=1/2$. As $t \rightarrow \infty$, all trajectories approach one of the four limit cycles, which are plotted as the black dashed lines. As in Fig. 2, the black dots denote the final position of each calculated trajectory at time $t=100$. **b** Twenty illustrative trajectories of the ODE system (53–55) for $K=9$, the parameter choices $a_1 = b_1 = a_2 = b_3 = a_6 = b_7 = 2$, $a_3 = b_2 = a_4 = b_4 = a_5 = b_8 =6$, $a_7 = a_8 = a_9 = b_5 = b_6 = b_9 = 10$, $\varepsilon =1$ and the initial condition (56). As $t \rightarrow \infty$, all trajectories approach one of the nine limit cycles, which are plotted as the black dashed lines. The black dots denote the final position of each calculated trajectory at time $t=100$ (Color figure online)

The illustrative dynamics of the ODE system (53–55) is presented in Fig. 3a, where we use the same parameters as we use in Fig. 2b for the ODE system (44–46). We observe that the presented trajectories converge to one of the four limit cycles as in Fig. 2b. The shape of the limit cycles is slightly modified by using the *x*-factorable transformation, but the limit cycles are still there as we formally prove in Sect. 6.

The *x*-factorable transformations modify the dynamics on the *x*-axis and *y*-axis. In Fig. 3a, we present illustrative trajectories which all start with the positive values of *x*(0) and *y*(0), while in Fig. 2b, some of the illustrative trajectories have zero initial values of *x*(0) and *y*(0). To get a comparable plot, we use the same initial conditions in both Fig. 2b and Fig. 3a, with the only exception that all initial conditions with $x(0)=0$ (resp. $y(0)=0$) in Fig. 2(b) are replaced by $x(0)=1/2$ (resp. $y(0)=1/2$) in Fig. 3a. We note that if we start a trajectory of the ODE system (53–55) on the *x*-axis or the *y*-axis, then it stays on the axis.

In Fig. 3b, we present illustrative dynamics of the ODE system (53–55) for $K=9$, showing that each computed trajectory converges to one of the 9 limit cycles denoted by black dashed lines. To illustrate that this behavior does not require special choices of initial conditions, we used different initial conditions for *x*(0) and *y*(0) together with the initial conditions for variables $v_i$ satisfying

56 $$\begin{aligned} v_i(0) = 1, \qquad \text{ for } \; i=1,2,\dots ,K. \end{aligned}$$

However, a similar figure can be obtained if we replace (56) with the initial condition (43), or if we initialize all values of $v_i$, $i=1,2,\dots ,K$ as zero (results not shown).

A CRN corresponding to reaction rate equations (53–55) can be obtained (by applying the construction in the proof of Lemma 1) as the CRN with $K+2$ chemical species, i.e., using the notation in Definition 1, we have

57 $$\begin{aligned} {\mathcal {S}} = \left\{ X, Y, V_1, V_2, \dots , V_K \right\} . \end{aligned}$$

To specify the reaction complexes and chemical reactions, we expand the right-hand side of reaction rate equations (53–55). First, we rewrite ODEs (55) as

58 $$\begin{aligned} \frac{\text{ d }v_i}{\text{ d }t}&= - k_{i,1} \, v_i + k_{i,2} \, v_i x + k_{i,3} \, v_i y - k_{i,4} \, v_i x^2 - k_{i,5} \, v_i y^2 + k_{i,6} \, v_i x^3 + k_{i,7} \, v_i y^3 \nonumber \\&\quad - k_{i,8} \, v_i x^4 - k_{i,9} \, v_i y^4 + k_{i,10} \, v_i x^5 + k_{i,11} \, v_i y^5 - v_i x^6 / \varepsilon - v_i y^6 / \varepsilon + 1 / \varepsilon , \qquad \end{aligned}$$

where $k_{i,j}$, $i=1,2,\dots ,K,$ $j=1,2,\dots ,11,$ are positive constants given by

59 $$\begin{aligned} \begin{aligned} k_{i,1}&= (1 + a_i^6 + b_i^6) / \varepsilon , \quad k_{i,2} = 6 a_i^5 / \varepsilon , \quad k_{i,3} = 6 b_i^5 / \varepsilon , \quad k_{i,4} = 15 a_i^4 / \varepsilon ,\\ k_{i,5}&= 15 b_i^4 / \varepsilon , \quad k_{i,6} = 20 a_i^3 / \varepsilon , \quad k_{i,7} = 20 b_i^3 / \varepsilon , \quad k_{i,8} = 15 a_i^2 / \varepsilon , \; \\ k_{i,9}&= 15 b_i^2 / \varepsilon , \quad k_{i,10} = 6 a_i / \varepsilon \quad \text{ and } \quad k_{i,11} = 6 b_i / \varepsilon . \end{aligned} \end{aligned}$$

Consequently, the right-hand side of Eq. (55) can be interpreted as the set of 14 chemical reactions for each $i=1,2,\dots ,K$. We define it as

60 $$\begin{aligned} {\mathcal {R}}_i \!= & {} \! \left\{ V_i \mathop {\longrightarrow }^{k_{i,1}} \emptyset , \quad V_i+X \mathop {\longrightarrow }^{k_{i,2}} 2V_i+X, \quad V_i+Y \mathop {\longrightarrow }^{k_{i,3}} 2V_i+Y, \quad V_i+2X \mathop {\longrightarrow }^{k_{i,4}} 2X, \right. \nonumber \\{} & {} \;\; V_i+2Y \mathop {\longrightarrow }^{k_{i,5}} 2Y, \qquad V_i+3X \mathop {\longrightarrow }^{k_{i,6}} 2V_i+3X, \qquad V_i+3Y \mathop {\longrightarrow }^{k_{i,7}} 2V_i+3Y, \qquad \;\; \nonumber \\{} & {} \;\; V_i+4X \mathop {\longrightarrow }^{k_{i,8}} 4X, \qquad V_i+4Y \mathop {\longrightarrow }^{k_{i,9}} 4Y, \qquad V_i+5X \mathop {\longrightarrow }^{k_{i,10}} 2V_i+5X, \nonumber \\{} & {} \left. \;\; V_i+5Y \mathop {\longrightarrow }^{k_{i,11}} 2V_i+5Y, \quad V_i+6X \mathop {\longrightarrow }^{1 / \varepsilon } 6X, \quad V_i+6Y \mathop {\longrightarrow }^{1 / \varepsilon } 6Y, \quad \emptyset \mathop {\longrightarrow }^{1 / \varepsilon } V_i \right\} .\nonumber \\ \end{aligned}$$

Consequently, reaction rate equation (55) corresponds to $14\, K$ chemical reactions in sets ${\mathcal {R}}_i$, $i=1,2,\dots ,K.$ Similarly, we rewrite ODEs (53–54) as

61 $$\begin{aligned} \frac{\text{ d }x}{\text{ d }t} \!= & {} \! \sum _{i=1}^K \bigg [- v_i x^4 + k_{i,12} \, v_i x^3 - k_{i,13} \, v_i x^2 + k_{i,14} \, v_i x + a_i \, v_i x y^2 \nonumber \\{} & {} \qquad + k_{i,15} \, v_i x^2 y - k_{i,16} \, v_i x y - \, v_i x^2 y^2\bigg ], \quad \; \end{aligned}$$

62 $$\begin{aligned} \frac{\text{ d }y}{\text{ d }t} \!= & {} \! \sum _{k=1}^K \bigg [- v_i y^4 + k_{i,17} \, v_i y^3 - k_{i,18} \, v_i y^2 + k_{i,19} \, v_i y + b_i \, v_i x^2 y \nonumber \\{} & {} \qquad + k_{i,20} \, v_i x y^2 - k_{i,21} \, v_i x y - \, v_i x^2 y^2 \bigg ], \quad \; \end{aligned}$$

where $k_{i,j}$, $i=1,2,\dots ,K,$ $j=12,13,\dots ,21,$ are constants given by

63 $$\begin{aligned}&k_{i,12} = 3 a_i, \quad k_{i,13} = 3 a_i^2 + b_i^2 - 1, \quad k_{i,14} = a_i^3 + a_i b_i^2 + b_i - a_i, \quad k_{i,15} = 2 b_i, \nonumber \\&k_{i,16} = 1 + 2 a_i b_i, \quad k_{i,17} = 3 b_i, \quad k_{i,18} = a_i^2 + 3 b_i^2 - 1,\nonumber \\&k_{i,19} = b_i^3 + a_i^2 b_i - a_i - b_i, \quad k_{i,20} = 2 a_i, \quad k_{i,21} = 2 a_i b_i - 1. \end{aligned}$$

Considering sufficiently large $a_i$ and $b_i$ (say, for $a_i>1$ and $b_i>1$), the constants (63) are positive. Moreover, since the term $- v_i x^2 y^2$ appears in both Eqs. (61) and (62), the right-hand sides of Eqs. (53–54) can be interpreted as the set of $15 \, K$ chemical reactions. We define

64 $$\begin{aligned} {\mathcal {R}}_i^* \!= & {} \! \left\{ V_i + 4X \mathop {\longrightarrow }^{1} V_i + 3X, \quad V_i + 3X \mathop {\longrightarrow }^{k_{i,12}} V_i + 4X, \quad V_i + 2X \mathop {\longrightarrow }^{k_{i,13}} V_i + X, \right. \nonumber \\{} & {} \;\; V_i + X \mathop {\longrightarrow }^{k_{i,14}} V_i + 2X, \qquad V_i + X + 2Y \mathop {\longrightarrow }^{a_i} V_i + 2X + 2Y, \nonumber \\{} & {} \;\; V_i + 2X + Y \mathop {\longrightarrow }^{k_{i,15}} V_i + 3X +Y, \qquad V_i + X + Y \mathop {\longrightarrow }^{k_{i,16}} V_i + Y, \nonumber \\{} & {} \;\; V_i + 2X + 2Y \mathop {\longrightarrow }^{1} V_i + X + Y, \qquad V_i + 4Y \mathop {\longrightarrow }^{1} V_i + 3Y, \nonumber \\{} & {} \;\; V_i + 3Y \mathop {\longrightarrow }^{k_{i,17}} V_i + 4Y, \quad V_i + 2Y \mathop {\longrightarrow }^{k_{i,18}} V_i + Y, \quad V_i + Y \mathop {\longrightarrow }^{k_{i,19}} V_i + 2Y, \nonumber \\{} & {} \;\; V_i + 2X + Y \mathop {\longrightarrow }^{b_i} V_i + 2X + 2Y, \qquad V_i + X + 2Y \mathop {\longrightarrow }^{k_{i,20}} V_i + X + 3Y, \nonumber \\{} & {} \left. \;\; V_i + X + Y \mathop {\longrightarrow }^{k_{i,21}} V_i + X \right\} , \qquad \text{ for } \quad i=1,2,\dots ,K. \end{aligned}$$

Then, we conclude that the reaction rate Eqs. (53–55) correspond to the CRN with $N=K+2$ chemical species and $29 \, K$ chemical reactions of at most seventh order given by

65 $$\begin{aligned} {\mathcal {R}} = \bigcup _{i=1}^K {\mathcal {R}}_i \cup {\mathcal {R}}_i^*. \end{aligned}$$

The CRN $({\mathcal {S}},{\mathcal {C}},{\mathcal {R}})$ consisting of chemical species ${\mathcal {S}}$ given by (57) and chemical reactions ${\mathcal {R}}$ given by (65) is the CRN which we will use to prove Theorem 1 in Sect. 6. The corresponding set of reaction complexes ${\mathcal {C}}$ can be inferred from the provided lists of reactions ${\mathcal {R}}_i$ and ${\mathcal {R}}_i^*$, $i=1,2,\dots ,K$, given by (60) and (64).

## Proof of Theorem 1

### Theorem 1

Let *K* be an arbitrary positive integer. Then there exists a CRN with *N*(*K*) chemical species which are subject to *M*(*K*) chemical reactions of at most seventh order such that

(i) Reaction rate equations (3) have at least *K* stable limit cycles,
(ii) We have $N(K) \le K+2$ and $M(K) \le 29 \, K$.

The idea of the proof of Theorem 1 is similar to the one chosen in Sects. 3 and 4, where we have first proven Lemma 2 about the existence of *K* limit cycles in the planar ODE system (10–11) and then we have used it to prove the existence of *K* limit cycles in the $(K+2)$-dimensional ODE system in Lemma 3. In this section, we will again start by formulating Lemma 4 for a planar ODE system, establishing that it has *K* stable limit cycles. This is again proven by using *K* disjoint sets (15). The result of Lemma 4 is then used in Lemma 5 to prove Theorem 1 by considering the $(K+2)$-dimensional ODE system (53–55).

The planar ODE system in Lemma 4 is derived by applying the *x*-factorable transformation to the planar ODE system (10–11). We obtain

66 $$\begin{aligned} \frac{\text{ d }x}{\text{ d }t} \!= & {} \! \sum _{k=1}^K x\,\,f_k(x-a_k,y-b_k) \!=\! x\,\,f(x,y) , \quad \; \end{aligned}$$

67 $$\begin{aligned} \frac{\text{ d }y}{\text{ d }t} \!= & {} \! \sum _{k=1}^K y\,\,g_k(x-a_k,y-b_k) \!=\! y\,\,g(x,y) , \quad \; \end{aligned}$$

where we have used the notation $f_k(\cdot ,\cdot )$ and $g_k(\cdot ,\cdot )$ introduced in Eqs. (18), (19) and (22).

The dynamics of the ODE system (66–67) is similar to the dynamics of the original planar ODE system (10–11) in the same way as the dynamics of the $(K+2)$-dimensional extended ODE system (53–55) is similar to the dynamics of the $(K+2)$-dimensional extended ODE system (44–46). We have already observed in Fig. 3(a) that the limit cycle around the point $(a_i,b_i)=(6,6)$ of the ODE system (44–46) is relatively circular. On the other hand, the shape of the limit cycles can more significantly differ between Figs. 2b and 3a if the corresponding parameters $a_i$ and $b_i$ are not equal to each other. Motivated by this observation, we will study the case $a_i = b_i$ in Lemma 4 and prove that it is possible to choose these parameters in a way that the planar ODE system (66–67) have (at least) *K* stable limit cycles. This result is sufficient for the proof of Theorem 1. However, we also note that the existence of limit cycles of the ODE system (66–67) is not restricted to the case $a_i = b_i$ and a more general lemma could be stated and proven, as we did in Lemma 2 where the existence of *K* limit cycles has been proven under a relatively general condition (14). The advantage of the case $a_i = b_i$ is that it simplifies the proof of Lemma 4, because we can use the approach and notations introduced in the proof of Lemma 2.

### Lemma 4

Let us assume that

68 $$\begin{aligned} a_i= b_i=8 {\hspace{0.56905pt}} i {\hspace{0.42677pt}} K \end{aligned}$$

for $i=1,2,\dots ,K.$ Then the ODE system (66–67) has at least *K* stable limit cycles.

### Proof

Let us define regions $\Omega _i \subset {{\mathbb {R}}}^2$, $i=1,2,\dots ,K,$ together with their boundary parts $\partial \Omega _{i1}$ and $\partial \Omega _{i2}$ by (15), (16) and (17). Our choice of values of $a_i$ and $b_i$ in (68) satisfies the assumption (14) in Lemma 2. Therefore, the ODE system (10–11) has with parameters given by (68) at least *K* stable limit cycles. Moreover, in the proof of Lemma 2, we have shown that each region $\Omega _i$ does not include any equilibrium of the planar ODE system (10–11). Any equilibrium of the ODE system (66–67) is either located on the *x*-axis or *y*-axis, or it is also an equilibrium of the ODE system (10–11). However, our assumption (68) implies that no region $\Omega _i$, $i=1,2,\dots ,K,$ intersects with the *x*-axis or *y*-axis. Therefore, we conclude that each $\Omega _i$, for $i=1,2,\dots ,K$, does not contain any equilibrium of the ODE system (66–67). Next, consider any point $(x_b,y_b)\in \partial \Omega _i$. We will compute the scalar product of vectors

69 $$\begin{aligned} (x_b-a_i,y_b-b_i) \; \text{ and } \; \big (x_b\,f(x_b,y_b), y_b\,g(x_b,y_b)\big ) \end{aligned}$$

by rewriting the second vector as a sum of two vectors

70 $$\begin{aligned} \big (x_b\,f(x_b,y_b), y_b\,g(x_b,y_b)\big ) = x_b \big (f(x_b,y_b),g(x_b,y_b)\big ) + \big (0, (y_b-x_b) \, g(x_b,y_b) \big ). \nonumber \\ \end{aligned}$$

The scalar product of vectors

71 $$\begin{aligned} (x_b-a_i,y_b-b_i) \; \text{ and } \; x_b \big (f(x_b,y_b), g(x_b,y_b)\big ) \end{aligned}$$

has already been calculated in the proof of Lemma 2 starting with Eq. (33). We obtained that it is negative for $(x_b,y_b)\in \partial \Omega _{i1}$ and positive for $(x_b,y_b)\in \partial \Omega _{i2}$. Therefore, the vector $x_b \big (f(x_b,y_b), g(x_b,y_b)\big )$ always points inside the domain $\Omega _i$ on all parts of the boundary $\partial \Omega _i.$ Next, we want to show that this conclusion also holds if vector $x_b \big (f(x_b,y_b), g(x_b,y_b)\big )$ is modified by adding the vector $\big (0, (y_b-x_b) \, g(x_b,y_b) \big )$ as it is done in Eq. (70). To do this, we note that our choice of parameters (68) implies that

$$\begin{aligned} (a_i-a_j)^2+(b_i-b_j)^2 = 128 {\hspace{0.56905pt}} (i-j)^2 K^2 \end{aligned}$$

for all $i,j=1,2,\dots ,K$, which not only satisfies the assumption (14), but it can be used in Eq. (37) to make a stronger conclusion that the scalar product of vectors (71) is at most $-1.45$ for $(x_b,y_b)\in \partial \Omega _{i1}$ and at least 1.45 for $(x_b,y_b)\in \partial \Omega _{i2}$. Thus, we only need to show that the scalar product of vectors

72 $$\begin{aligned} (x_b-a_i,y_b-b_i) \qquad \text{ and } \qquad \big (0, (y_b-x_b) \, g(x_b,y_b) \big ) \end{aligned}$$

is in absolute value less than 1.45 to conclude that the original scalar product (69) is negative for $(x_b,y_b)\in \partial \Omega _{i1}$ and positive for $(x_b,y_b)\in \partial \Omega _{i2}$. Using the definition of $g(\cdot ,\cdot )$ in (22) and the notation $z_1=x_b-a_i$, $z_2=y_b-b_i$ introduced in the proof of Lemma 2, we have $y_b-x_b=z_2-z_1$ and the scalar product (72) can be written as

73 $$\begin{aligned} (z_2-z_1)\,z_2\,\, g_i(z_1,z_2) + (z_2-z_1) \, z_2 \!\!\!\! \sum _{k=1, k \ne i}^K \!\! g_k(x_b-a_k,y_b-a_k). \end{aligned}$$

Since we have

$$\begin{aligned} \max _{(x_b,y_b)\in \partial \Omega _i} \big \vert (z_2-z_1)\,z_2\,\, g_i(z_1,z_2) \big \vert = \max _{z_1^2+z_2^2=2 \; (\text{ or } \; 1/2)} \big \vert (z_2-z_1)\,z_2\,\, g_i(z_1,z_2) \big \vert \le 1.4 \end{aligned}$$

and the second term in (73) is also less than 0.05, we conclude that the scalar product (69) is negative for $(x_b,y_b)\in \partial \Omega _{i1}$ and positive for $(x_b,y_b)\in \partial \Omega _{i2}$. Therefore, the vector $\big (x_b\,f(x_b,y_b), y_b\,g(x_b,y_b)\big )$ always points inside the domain $\Omega _i$ on all parts of the boundary $\partial \Omega _i.$ In particular, applying Poincaré-Bendixson theorem (Strogatz 2015), we conclude that each $\Omega _i$ contains at least one stable limit cycle. Since $\Omega _i$, $i=1,2,\dots ,K,$ are pairwise disjoint, this implies that the ODE system (66–67) has at least *K* stable limit cycles. $\square$

The following lemma shows that the extended ODE system (53–55) has at least *K* stable limit cycles when $\varepsilon$ is small enough. The detailed proof is omitted since one can use similar steps as in the proof of Lemma 3.

### Lemma 5

Let us assume that constants $a_i,$ $b_i$, $i=1,2,\dots ,K$ are given by (68). Then there exists $\varepsilon _0>0$ such that the reaction rate equations (53)–(55) have at least *K* stable limit cycles for all $\varepsilon \in (0,\varepsilon _0).$

### Proof

This follows directly from Lemma 4 and Tikhonov’s theorem (Tikhonov 1952; Klonowski 1983). $\square$

The existence of *K* limit cycles in the CRN (65) follows by application of Lemma 5. The Chemical system (65) has $(K+2)$ chemical species *X*,  *Y*,  $V_1$, $V_2$, ..., $V_K$, which are subject to 29*K* chemical reactions, so, by construction, we also establish bounds in part (ii) of Theorem 1 on *N*(*K*) and *M*(*K*). This concludes the proof of Theorem 1.

## Proof of Theorem 2

### Theorem 2

Let *K* be an arbitrary positive integer. Then there exists a CRN with *N*(*K*) chemical species which are subject to *M*(*K*) chemical reactions of at most second order such that

(i) Reaction rate equations (3) have at least *K* stable limit cycles,
(ii) We have $N(K) \le 7K+14$ and $M(K) \le 42 \, K + 24$.

In Theorem 1, we have established that the reaction rate equations (53–55) describing the CRN (65) have at least *K* stable limit cycles. Since the right-hand sides of ODEs (53–55) include polynomials up to the order 7, the resulting chemical reactions (65) are reactions of the order at most 7. However, in practice, every higher-order reactions can be subdivided into elementary steps, which are at most bimolecular (second order). Therefore, we focus here on the proof of Theorem 2 which restricts our considerations to at most second-order kinetics. We prove it by further extending the number of variables in the reaction rate equations (53–55), i.e., by adding intermediary chemical species and elementary reactions into the CRN (65). The resulting CRN has $N=7K+14$ chemical species denoted by

74 $$\begin{aligned} {\mathcal {S}} = \big \{ X, Y, W_1, W_2, \dots , W_{12} \big \} \cup \bigcup _{i=1}^K \left\{ V_i, Z_{i,1},Z_{i,2},Z_{i,3},Z_{i,4},Z_{i,5},Z_{i,6} \right\} , \end{aligned}$$

where we use the notation introduced in Definition 1 of CRNs. The concentrations *x*,  *y*,  $v_i$, $w_1$, $w_2$, $\dots$, $w_{12}$, $z_{i,j}$ for $i=1,2,\dots ,K$ and $j=1,2,\dots ,6$ evolve according to reaction rate equations

75 $$\begin{aligned} \frac{\text{ d }x}{\text{ d }t} \!= & {} \! \sum _{i=1}^K \bigg [- x z_{i,3} + k_{i,12} \, v_i w_2 - k_{i,13} \, x z_{i,1} + k_{i,14} \, v_i x + a_i \, v_i w_{11} \nonumber \\{} & {} \qquad + k_{i,15} \, v_i w_{12} - k_{i,16} \, x z_{i,2} - \, x z_{i,5} \bigg ], \quad \; \end{aligned}$$

76 $$\begin{aligned} \frac{\text{ d }y}{\text{ d }t} \!= & {} \! \sum _{k=1}^K \bigg [- y z_{i,4} + k_{i,17} \, v_i w_7 - k_{i,18} \, y z_{i,2} + k_{i,19} \, v_i y + b_i \, v_i w_{12} \nonumber \\{} & {} \qquad + k_{i,20} \, v_i w_{11} - k_{i,21} \, y z_{i,1} - \, y z_{i,6} \bigg ], \quad \; \end{aligned}$$

77 $$\begin{aligned} \frac{\text{ d }v_i}{\text{ d }t} \!= & {} \! - k_{i,1} \, v_i + k_{i,2} \, v_i x + k_{i,3} \, v_i y - k_{i,4} \, v_i w_1 - k_{i,5} \, v_i w_6 \nonumber \\{} & {} \quad + \, k_{i,6} \, v_i w_2 + k_{i,7} \, v_i w_7 - k_{i,8} \, v_i w_3 - k_{i,9} \, v_i w_8 \end{aligned}$$

78 $$\begin{aligned}{} & {} \quad + \, k_{i,10} \, v_i w_4 + k_{i,11} \, v_i w_9 - v_i w_5 / \varepsilon - v_i w_{10} / \varepsilon + 1 / \varepsilon , \nonumber \\ \delta \, \frac{\text{ d }w_1}{\text{ d }t} \!= & {} \! x^2 - w_1, \qquad \delta \, \frac{\text{ d }w_2}{\text{ d }t} = x w_1 - w_2, \qquad \delta \, \frac{\text{ d }w_3}{\text{ d }t} = x w_2 - w_3, \end{aligned}$$

79 $$\begin{aligned} \delta \, \frac{\text{ d }w_4}{\text{ d }t} \!= & {} \! x w_3 - w_4, \qquad \delta \, \frac{\text{ d }w_5}{\text{ d }t} = x w_4 - w_5, \qquad \delta \, \frac{\text{ d }w_6}{\text{ d }t} = y^2 - w_6, \end{aligned}$$

80 $$\begin{aligned} \delta \, \frac{\text{ d }w_7}{\text{ d }t} \!= & {} \! y w_6 - w_7, \qquad \delta \, \frac{\text{ d }w_8}{\text{ d }t} = y w_7 - w_8, \qquad \delta \, \frac{\text{ d }w_9}{\text{ d }t} = y w_8 - w_9, \end{aligned}$$

81 $$\begin{aligned} \delta \, \frac{\text{ d }w_{10}}{\text{ d }t} \!= & {} \! y w_9 - w_{10}, \quad \; \delta \, \frac{\text{ d }w_{11}}{\text{ d }t} = x w_6 - w_{11}, \quad \; \delta \, \frac{\text{ d }w_{12}}{\text{ d }t} = y w_1 - w_{12}, \end{aligned}$$

82 $$\begin{aligned} \delta \, \frac{\text{ d }z_{i,1}}{\text{ d }t} \!= & {} \! v_i x - z_{i,1}, \qquad \delta \, \frac{\text{ d } z_{i,2}}{\text{ d }t} = v_i y - z_{i,2}, \qquad \delta \, \frac{\text{ d } z_{i,3}}{\text{ d }t} = v_i w_2 - z_{i,3}, \end{aligned}$$

83 $$\begin{aligned} \delta \, \frac{\text{ d } z_{i,4}}{\text{ d }t} \!= & {} \! v_i w_7 - z_{i,4}, \quad \delta \, \frac{\text{ d } z_{i,5}}{\text{ d }t} = v_i w_{11} - z_{i,5}, \quad \delta \, \frac{\text{ d } z_{i,6}}{\text{ d }t} = v_i w_{12} - z_{i,6}, \quad \end{aligned}$$

where $\delta >0$, $\varepsilon >0$ and $k_{i,j}$, $i=1,2,\dots ,K,$ $j=1,2,\dots ,21,$ are positive constants given by (59) and (63). Considering the limit $\delta \rightarrow 0$ in Eqs. (78–83), we obtain

84 $$\begin{aligned} \begin{aligned}&\qquad w_1 = x^2, \quad w_2 = x^3, \quad w_3 = x^4, \quad w_4 = x^5, \quad w_5 = x^6, \quad w_6 = y^2,\\&\quad w_7 = y^3, \quad w_8 = y^4, \quad w_9 = y^5, \quad w_{10} = y^6, \quad w_{11} = x y^2, \quad w_{12} = x^2 y, \\&z_{i,1} = v_i x, \quad z_{i,2} = v_i y, \quad z_{i,3} = v_i x^3 \!, \quad z_{i,4} = v_i y^3 \!, \quad z_{i,5} = v_i x y^2 \!, \quad z_{i,6} = v_i x^2 y. \end{aligned} \end{aligned}$$

Substituting the limiting values (84) for $w_\ell$ and $z_{i,j}$, $\ell =1,2,\dots ,12,$ $i=1,2\dots ,K$, $j=1,2,\dots ,6$, into Eqs. (75–77), we obtain Eqs. (61), (62) and (58), which are equal to the reaction rate equations (53–55). In particular, we deduce the following lemma, establishing that the extended ODE system (75–83) with $N=7K+14$ variables has at least *K* stable limit cycles when $\delta$ and $\varepsilon$ are small enough.

### Lemma 6

Let us assume that constants $a_i,$ $b_i$, $i=1,2,\dots ,K$ are given by (68). Then there exist $\delta _0>0$ and $\varepsilon _0>0$ such that reaction rate equations (75–83) have at least *K* stable limit cycles for all $\delta \in (0,\delta _0)$ and $\varepsilon \in (0,\varepsilon _0).$

### Proof

This follows directly from Lemma 5 and Tikhonov’s theorem (Tikhonov 1952; Klonowski 1983). $\square$

The right-hand sides of reaction rate equations (75–83) only include quadratic terms. Therefore, there exists a CRN corresponding to the reaction rate Eqs. (75–83) which includes (at most) second-order reactions. We can obtain it by applying the construction in the proof of Lemma 1. The right-hand sides of Eqs. (75) and (76) can be interpreted as the set of $16 \, K$ chemical reactions (compare with (64) for ODEs (53–54))

85 $$\begin{aligned} {\mathcal {R}}_i^{s,*} \!= & {} \! \left\{ X + Z_{i,3} \mathop {\longrightarrow }^{1} Z_{i,3}, \quad V_i + W_2 \mathop {\longrightarrow }^{k_{i,12}} V_i + W_2 + X, \quad X + Z_{i,1} \mathop {\longrightarrow }^{k_{i,13}} Z_{i,1}, \right. \nonumber \\{} & {} \;\; V_i + X \mathop {\longrightarrow }^{k_{i,14}} V_i + 2X, \quad V_i + W_{11} \mathop {\longrightarrow }^{a_i} V_i + W_{11} + X, \nonumber \\{} & {} \;\; V_i + W_{12} \mathop {\longrightarrow }^{k_{i,15}} V_i + W_{12} + X, \quad X + Z_{i,2} \mathop {\longrightarrow }^{k_{i,16}} Z_{i,2}, \nonumber \\{} & {} \;\; X + Z_{i,5} \mathop {\longrightarrow }^{1} Z_{i,5}, \quad Y + Z_{i,6} \mathop {\longrightarrow }^{1} Z_{i,6}, \quad Y + Z_{i,4} \mathop {\longrightarrow }^{1} Z_{i,4}, \nonumber \\{} & {} \;\; V_i + W_{7} \mathop {\longrightarrow }^{k_{i,17}} V_i + W_7 + Y, \quad Y + Z_{i,2} \mathop {\longrightarrow }^{k_{i,18}} Z_{i,2}, \quad V_i + Y \mathop {\longrightarrow }^{k_{i,19}} V_i + 2Y, \nonumber \\{} & {} \;\; V_i + W_{12} \mathop {\longrightarrow }^{b_i} V_i + W_{12} + Y, \quad V_i + W_{11} \mathop {\longrightarrow }^{k_{i,20}} V_i + W_{11} + Y, \nonumber \\{} & {} \left. \;\; Y + Z_{i,1} \mathop {\longrightarrow }^{k_{i,21}} Z_{i,1} \right\} , \quad \text{ for } \quad i=1,2,\dots ,K. \end{aligned}$$

The right-hand side of equations (77) can be interpreted as the set of 14 chemical reactions for each $i=1,2,\dots ,K$ (compare with (60) for the right-hand side of ODE (55))

86 $$\begin{aligned} {\mathcal {R}}_i^{s} \!= & {} \! \left\{ V_i \mathop {\longrightarrow }^{k_{i,1}} \emptyset , \quad V_i+X \mathop {\longrightarrow }^{k_{i,2}} 2V_i+X, \quad V_i+Y \mathop {\longrightarrow }^{k_{i,3}} 2V_i+Y, \right. \nonumber \\{} & {} \quad V_i+W_1 \mathop {\longrightarrow }^{k_{i,4}} W_1, \quad V_i+W_6 \mathop {\longrightarrow }^{k_{i,5}} W_6, \qquad V_i+W_2 \mathop {\longrightarrow }^{k_{i,6}} 2V_i+W_2, \nonumber \\{} & {} \quad V_i+W_7 \mathop {\longrightarrow }^{k_{i,7}} 2V_i+W_7, \quad V_i+W_3 \mathop {\longrightarrow }^{k_{i,8}} W_3, \quad V_i+W_8 \mathop {\longrightarrow }^{k_{i,9}} W_8, \nonumber \\{} & {} \quad V_i+W_4 \mathop {\longrightarrow }^{k_{i,10}} 2V_i+W_4, \quad V_i+W_9 \mathop {\longrightarrow }^{k_{i,11}} 2V_i+W_9, \nonumber \\{} & {} \left. \quad \! V_i+W_5 \mathop {\longrightarrow }^{1 / \varepsilon } W_5, \quad V_i+W_{10} \mathop {\longrightarrow }^{1 / \varepsilon } W_{10}, \quad \emptyset \mathop {\longrightarrow }^{1 / \varepsilon } V_i \right\} . \end{aligned}$$

Consequently, reaction rate equations (75–77) correspond to $30\, K$ chemical reactions in sets ${\mathcal {R}}_i^{s,*}$ and ${\mathcal {R}}_i^s$, $i=1,2,\dots ,K.$ This is already more than $29 \, K$ chemical reactions used in Theorem 1, because we did not combine two terms on the right-hand sides into one reaction as we did in the set ${\mathcal {R}}_i^{*}$ (this is further discussed in Eq. (92) in Sect. 9). Moreover, there are additional chemical reactions corresponding to the dynamics of additional chemical species in Eqs. (78–83). The right-hand sides of equations (78–81) can be interpreted as the set of 24 chemical reactions given as

87 $$\begin{aligned} {\mathcal {R}}^{w} \!= & {} \! \left\{ 2 X \mathop {\longrightarrow }^{1/\delta } 2X + W_1, \quad 2 Y \mathop {\longrightarrow }^{1/\delta } 2 Y + W_6, \right. \nonumber \\{} & {} \quad X + W_j \mathop {\longrightarrow }^{1/\delta } X + W_j + W_{j+1}, \quad \text{ for } \;\; j=1,2,3,4, \nonumber \\{} & {} \quad Y + W_j \mathop {\longrightarrow }^{1/\delta } Y + W_j + W_{j+1}, \quad \text{ for } \;\; j=6,7,8,9, \nonumber \\{} & {} \quad X + W_6 \mathop {\longrightarrow }^{1/\delta } X + W_6 + W_{11}, \quad Y + W_1 \mathop {\longrightarrow }^{1/\delta } Y + W_1 + W_{12}, \nonumber \\{} & {} \left. \quad W_\ell \mathop {\longrightarrow }^{1/\delta } \emptyset , \quad \text{ for } \;\; \ell = 1,2,\dots ,12 \right\} . \end{aligned}$$

Finally, the right-hand sides of Eqs. (82–83) can be interpreted as the set of 12 chemical reactions for each $i=1,2,\dots ,K$ given by

88 $$\begin{aligned} {\mathcal {R}}_i^{z} \!= & {} \! \left\{ X + V_i \mathop {\longrightarrow }^{1/\delta } X + V_i + Z_{i,1}, \quad Y + V_i \mathop {\longrightarrow }^{1/\delta } Y + V_i + Z_{i,2}, \right. \nonumber \\{} & {} \quad V_i + W_2 \mathop {\longrightarrow }^{1/\delta } V_i + W_2 + Z_{i,3}, \quad V_i + W_7 \mathop {\longrightarrow }^{1/\delta } V_i + W_7 + Z_{i,4}, \nonumber \\{} & {} \quad V_i + W_{11} \mathop {\longrightarrow }^{1/\delta } V_i + W_{11} + Z_{i,5}, \quad V_i + W_{12} \mathop {\longrightarrow }^{1/\delta } V_i + W_{12} + Z_{i,6}, \nonumber \\{} & {} \left. \quad Z_{i,j} \mathop {\longrightarrow }^{1/\delta } \emptyset , \quad \text{ for } \;\; j = 1,2,\dots ,6 \right\} . \end{aligned}$$

In summary, we conclude that the reaction rate equations (75–83) correspond to the CRN with $N=7K+14$ chemical species and $42 \, K + 24$ chemical reactions given by

89 $$\begin{aligned} {\mathcal {R}} = {\mathcal {R}}^w \cup \bigcup _{i=1}^K {\mathcal {R}}_i^s \cup {\mathcal {R}}_i^{s,*} \cup {\mathcal {R}}_i^z. \end{aligned}$$

Using Lemma 6, we deduce that the CRN $({\mathcal {S}},{\mathcal {C}},{\mathcal {R}})$ consisting of chemical species ${\mathcal {S}}$ given by (74) and chemical reactions ${\mathcal {R}}$ given by (89) is an example of a CRN which satisfies Theorem 2. The corresponding set of reaction complexes ${\mathcal {C}}$ can be inferred from the provided lists of reactions ${\mathcal {R}}_i^{s,*}$, ${\mathcal {R}}_i^s$, ${\mathcal {R}}^w$ and ${\mathcal {R}}_i^z$, for $i=1,2,\dots ,K$, given by (85), (86), (87) and (88).

## Proof of Theorem 3

### Theorem 3

Let *C*(*n*) be the maximum number of stable limit cycles of reaction rate equations (1–2) corresponding to CRNs with two chemical species undergoing chemical reactions of at most *n*-th order. Then we have

4 $$\begin{aligned} C(n) \ge \left\lfloor \frac{n+2}{6} \right\rfloor , \end{aligned}$$

where the floor function $\lfloor . \rfloor$ denotes the integer part of a positive real number.

Given an arbitrarily large integer $K \in {{\mathbb {N}}}$, we will show that there exists a CRN with two chemical species such that its reaction rate equations have at least *K* stable limit cycles and the order of the chemical reactions is at most $n(K)=6K-2$. To do that, we start with the planar ODE system  (10–11) and renormalize time *t* to get a planar system with polynomial ODEs. Using an auxiliary function

$$\begin{aligned} h(x,y) = \prod _{k=1}^K \Big ( 1 + (x-a_k)^6 + (y-b_k)^6 \Big ), \end{aligned}$$

we define our new time variable $\tau$ by

$$\begin{aligned} \tau = \int _0^t \frac{1}{h(x(s),y(s))} \, \text{ d } s. \end{aligned}$$

Then we obtain

90 $$\begin{aligned} \frac{\text{ d }x}{\text{ d }\tau } \!= & {} \! \frac{\text{ d }x}{\text{ d }t} \frac{\text{ d }t}{\text{ d }\tau } = h(x,y) \sum _{k=1}^K \frac{(x-a_k) \big \{1-(x-a_k)^2-(y-b_k)^2\big \} -(y-b_k)}{ 1 + (x-a_k)^6 + (y-b_k)^6} , \qquad \nonumber \\ \end{aligned}$$

91 $$\begin{aligned} \frac{\text{ d }y}{\text{ d }\tau } \!= & {} \! \frac{\text{ d }y}{\text{ d }t} \frac{\text{ d }t}{\text{ d }\tau } = h(x,y) \sum _{k=1}^K \frac{(y-b_k) \big \{1-(x-a_k)^2-(y-b_k)^2\big \} +(x-a_k)}{ 1 + (x-a_k)^6 + (y-b_k)^6} , \qquad \nonumber \\ \end{aligned}$$

which is a planar ODE system with its right-hand side given as polynomials of degree $n(K)-1=6K-3$. Since we only rescaled the time, Fig. 1(a) provides an illustrative dynamics of the ODE system (90–91) for $K=4$. The illustrative trajectories are calculated in Fig. 1a by solving ODEs (10–11) in time interval $t \in [0,100]$, and we can obtain the same result by solving ODEs (90–91) numerically in time interval $\tau \in [0, 10^{-9}]$. Applying *x*-factorable transformation to ODEs (90–91), we obtain

$$\begin{aligned} \frac{\text{ d }x}{\text{ d }\tau } \!= & {} \! x \, h(x,y) \sum _{k=1}^K \frac{(x-a_k) \big \{1-(x-a_k)^2-(y-b_k)^2\big \} -(y-b_k)}{ 1 + (x-a_k)^6 + (y-b_k)^6} , \quad \end{aligned}$$

$$\begin{aligned} \frac{\text{ d }y}{\text{ d }\tau } \!= & {} \! y \, h(x,y) \sum _{k=1}^K \frac{(y-b_k) \big \{1-(x-a_k)^2-(y-b_k)^2\big \} +(x-a_k)}{ 1 + (x-a_k)^6 + (y-b_k)^6} , \quad \end{aligned}$$

which is a kinetic system of ODEs with polynomials of degree $n(K)=6K-2$ and which has *K* stable limit cycles. Solving for *K*, we obtain $K=(n(K)+2)/6$, which establishes the lower bound (4) in Theorem 3.

## Discussion

The main results of this paper have been formulated as Theorems 1, 2 and 3, which show that there exist CRNs with *K* stable limit cycles for any integer $K \in {{\mathbb {N}}}.$ The CRN presented in our proof of Theorem 1 consisted of $N(K)=K+2$ chemical species ${\mathcal {S}}$ given by (57) and $M(K) = 29 \, K$ chemical reactions ${\mathcal {R}}$ (of at most seventh order) given by (65). The number of species and chemical reactions further increases in our proof of Theorem 2, where we restrict our investigation to CRNs with (at most) second-order kinetics. On the other hand, if we restrict to CRNs with only $N=2$ chemical species, then the order of the chemical reactions increases with *K* as $n(K)=6K-2$ in our proof of Theorem 3.

An important question is whether we can further decrease *N*(*K*) (the number of chemical species) and *M*(*K*) (the number of chemical reactions) in Theorems 1 and 2 and still obtain a CRN with *K* stable limit cycles. One possibility to decrease *M*(*K*) is to use one chemical reaction to interpret multiple terms on the right-hand sides of ODEs (53–55). We have already done this in the reaction set ${\mathcal {R}}_i^*$ given by (64) with the reaction

92 $$\begin{aligned} V_i + 2X + 2Y \mathop {\longrightarrow }^{1} V_i + X + Y, \end{aligned}$$

which corresponds to terms of the form $-v_i x^2 y^2$ appearing in both equations (53) and (54). Another way to construct a CRN with reactions modeling the two terms, $-v_i x^2 y^2$, in the reaction rate equations (53–54), is to use one chemical reaction per one term on the right-hand side. That is, the chemical reaction (92) could be replaced by two chemical reactions

$$\begin{aligned} V_i + 2X + 2Y \mathop {\longrightarrow }^{1} V_i + X + 2Y, \; \text{ and } \; V_i + 2X + 2Y \mathop {\longrightarrow }^{1} V_i + 2X + Y \end{aligned}$$

without modifying the form of the reaction rate equations (53–54). In particular, if our aim is to decrease the number *M*(*K*) of chemical reactions, we could consider to ‘merge’ some other reactions, which have the same reactants. For example, reaction lists (60) and (64) contain chemical reactions

$$\begin{aligned} V_i + 3Y \mathop {\longrightarrow }^{k_{i,7}} 2V_i+3Y, \quad V_i + 3Y \mathop {\longrightarrow }^{k_{i,17}} V_i + 4Y. \end{aligned}$$

If these chemical reactions had the same reaction rate constants $k_{i,7}$ and $k_{i,17}$, then we could replace them by one chemical reaction given by

$$\begin{aligned} V_i + 3Y \mathop {\longrightarrow }^{k_{i,7}} 2 V_i + 4Y \end{aligned}$$

and we would obtain a CRN which has $28 \, K$ chemical reactions rather than $29 \, K$, which we use in Theorem 1. Consequently, there is potential to decrease the size of the constructed CRN by a careful choice of our parameters or by modifying the right-hand sides of reaction rate equations (53–55). However, the focus of our paper was on the existence proofs and we leave the improvement of bounds on *N*(*K*) and *M*(*K*) to future work.

Another possible direction to investigate is to consider more detailed stochastic description of CRNs, written as continuous-time discrete space Markov chains and simulated by the Gillespie algorithm (Erban and Chapman 2020). Such simulations would help us to investigate how our parameters $a_i,$ $b_i$, $i=1,2,\dots ,K$, need to be chosen that the system not only has the limit cycles of comparable size (as we visualized in Fig. 3 in the ODE setting), but it also follows each of these limit cycles with a similar probability (comparable to 1/*K*). This could also be achieved by using the noise-control algorithm (Plesa et al. 2018) for designing CRNs. This algorithm structurally modifies a given CRN under mass-action kinetics, in such a way that (i) controllable state-dependent noise is introduced into the stochastic dynamics, while (ii) the reaction rate equations are preserved. In particular, it could be used to introduce additional chemical reactions (which do not change the ODE dynamics), but lead to controllable noise-induced switching between different limit cycles.

## Data Availability

Not applicable. No data are associated with this article.
